# Determination of blood dexmedetomidine in dried blood spots by LC-MS/MS to screen therapeutic levels in paediatric patients

**DOI:** 10.1371/journal.pone.0210391

**Published:** 2019-01-14

**Authors:** Liliana Rivera-Espinosa, Alejandra Toledo-López, Juan Luis Chávez-Pacheco, Radamés Alemón-Medina, Josefina Gómez-Garduño, Gustavo Lugo-Goytia, Raquel García-Álvarez, Hugo Juárez-Olguín, Luz María Torres-Espíndola, María-Gabriela Pérez-Guillé

**Affiliations:** 1 Pharmacology Laboratory, National Institute of Paediatrics, Mexico City, Mexico; 2 Anaesthesiology and Critical Medicine Department, National Institute of Medical Sciences and Nutrition, Mexico City, Mexico, Intensive Care Unit, National Institute of Respiratory Diseases, Mexico City, Mexico; H Lee Moffitt Cancer Center and Research Institute, UNITED STATES

## Abstract

Dexmedetomidine is an imidazole derivative, with high affinity for α2 adrenergic receptors, used for sedation, analgesia and adjuvant anaesthesia. In this study, an analytical method for the quantification of dexmedetomidine in dried blood spots was developed, validated and applied. The drug was extracted from dried blood spot by liquid extraction; the separation was carried out by ultra high-resolution liquid chromatography in reverse phase coupled to tandem mass spectrometry method. An X Select cyano 5 μm HSS column (2.1 X 150 mm, Waters) and a mobile phase composed of 0.1% formic acid: acetonitrile [50:50 v/v], were used. The test was linear over the concentration range of 50 to 2000 pg/mL. The coefficients of variation for the intra and interday trials were less than 15%. The drug was stable under the conditions tested. The method was successfully applied for the quantification of 6 patients, aged 0 to 2 years, with classification ASA I, who underwent ambulatory surgeries, receiving a dose of 1 μg/Kg dexmedetomidine IV. The drug concentrations in the different sampling times were in the range of 76 to 868 pg/mL.

## Introduction

Dexmedetomidine (DXM) is a drug from the group of the imidazoles (4-(S)-[1-(2,3-dimethylphenyl) ethyl]-1H-imidazole monohydrochloride), highly lipophilic and it is soluble in water [1]. DXM has a potent pure alpha adrenergic agonist 2 activity and highly selective, with a selectivity ratio of 1600: 1 (α2: α1) [2].

The Food and Drug Administration (FDA) of the United States approved its use in adults in 1999. It is used to obtain sedation, analgesia and as an adjunct to anaesthesia, decreasing the requirements of anaesthetic drugs in procedures requiring total intravenous anaesthesia [3–5].

Due to its lipophilic characteristics, it has a rapid distribution to the tissues (half-life of 6 min) and a rapid elimination (t_1/2_ 2–2.5 h), it is metabolized by cytochrome P450 isoenzymes, mainly CYP2A6 and is eliminated in the form of inactive metabolites, mainly glucuronides. It is excreted through urine (80–90%) and faeces (5–13%).

There is no evidence of clinically important drug interactions; however, it presents additive pharmacological effects when administered together with anaesthetics and opioid agonists, sedatives/hypnotics and vasodilators.

The administration of DXM helps to reduce the requirements of other anaesthetics. Like midazolam and propofol, it has sympatholytic properties and decreases the release of catecholamines. It has analgesic properties equal to those of morphine [6].

DXM causes few effects on respiratory function and is well tolerated in intensive therapy patients who are supported with mechanical ventilation [7–9].

The FDA has not approved the use of DXM in the paediatric population, so its use is conditioned by evidence-based medicine (administration of the dose reported for adults). Its use in paediatrics has recently increased, due to the fact that DXM does not interfere with respiratory function and is an excellent inducer of anaesthesia in surgical and dentistry procedures.

Currently there is a need for clinical and pharmacokinetic studies in paediatrics, which are useful to establish the optimal personalised dose; since the FDA has not approved it yet to be used in this population [10, 11]. In addition, other obstacles to perform such clinical trials have been the obtaining of the sample (venous puncture with catheter) and the amount of blood. In patients younger than 2 years, venous puncture is a very invasive procedure and the amount of sample is restricted.

To overcome this difficulty in the present work, we implemented a less invasive and less painful procedure, by obtaining a drop of blood (approximately 40 μL) from the heel or finger and collecting it on a Whatmann 903 filter paper card (Güthrie card), this procedure of blood collection is known as dried blood spot (DBS) and it offers the following advantages: minimally invasive, less painful, allows determination with lesser amounts of blood, allows therapeutic screening of drugs, permits their storage at room temperature and samples can be sent by mail without keeping very special conditions. Likely, it offers the alternative to avoid drug decomposition, since a number of degrading enzymes are denatured when the blood is dried within the filter paper. Also, it reduces the risk of viral disease transmission among researchers. Some compounds may require derivatization and/or stabilizing agents such as antioxidants, which can be placed before placing the sample to be analyzed. However, it shows a few disadvantages, since it requires very sensitive and expensive analytical techniques, it often requires more than a blood drop to perform repeats, a special training is required to obtain the samples, and there is a latent risk of contaminating the sample with additional amounts of drug when the same person prepares the dose and manipulates the blood samples [12].

The objective of this work was to develop, validate and implement an analytical method, based on ultra high-resolution liquid chromatography coupled to mass spectrometry (LC-MS/MS), capable of quantifying dexmedetomidine from samples in DBS. The validated method will be useful for pharmacokinetic studies or therapeutic monitoring.

## Material and methods

### Materials and reagents

The DXM standard (purity 99.9%) was purchased from Santa Cruz Biotechnology, and cyclophosphamide monohydrate (internal standard) from MP Biomedicals. (Fountain Pkwy, Solon OH, USA). Acetonitrile and methanol HPLC grade were obtained from EMD Millipore Co (Mexico). Ethyl acetate and formic acid reagent grade were purchased from Merck (Darmstat, Germany). Dichloroethane from Electron Microscopy Sciences (Washington, DC, USA).

The standards for selectivity were: ropivacaine, propofol, acetaminophen, fentanyl, which were purchased from MP Biomedicals (Fountain Pkwy, Solon OH, USA) and Toronto Research Chemicals Inc (Toronto, Canada). Milli-Q grade water (Millipore, Molsheim, France) was used to prepare all solutions and dilutions. Erythrocyte fraction (kept at 4°C) and blank plasma (frozen at -80°C), were obtained from the blood reservoir of our institution (healthy adult donors), having citrate phosphate dextrose as anticoagulant.

### Chromatographic conditions

The determination was made in an Acquity equipment (Waters, Milford MA, USA) ultra-performance liquid chromatography (UPLC) coupled to a Micromass Quattro Micro mass spectrometer (Waters Micromass, Manchester, UK). The equipment consists of column temperature control (adjusted to 40°C) and automatic autosampler (adjusted to 15°C).

The chromatographic separation was carried out on an X-Select HSS Cyano column (2.1 x 150 mm, 5 μm Waters). The mobile phase consisted in 0.1% formic acid in water: acetonitrile (50:50 v/v), flow rate of 0.3 mL/min and execution time of 3 min. The injection volume was 10 μL.

### Spectrometric conditions

**Fig 1:**
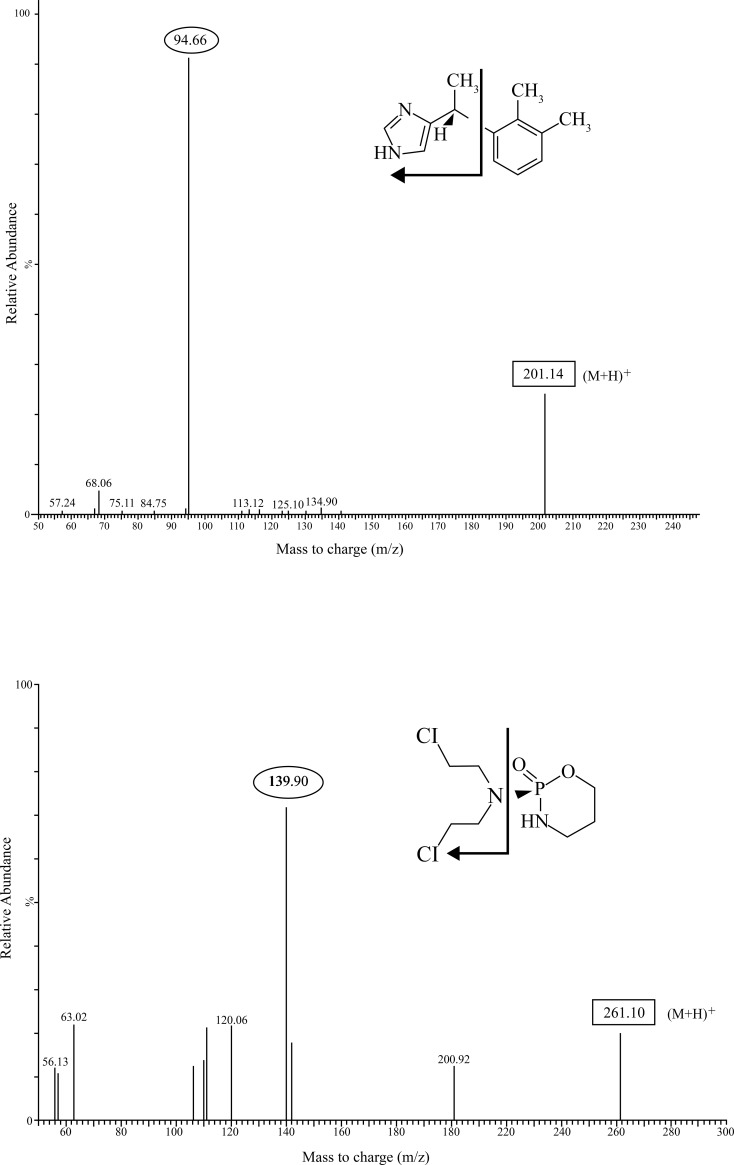
Fragmentation patterns of dexmedetomidine (upper panel) and internal standard (lower panel). The precursor ions are indicated in a box and product ion in an oval.

A mass spectrometer (Quattro Micro), with spray interface was operated in positive ion mode. Analytes of interest were measured in SRM mode, ion transitions were m/z1+ 201.14 > 94.66 for DXM and 261.00 > 139.90 for cyclophosphamide. Fig 1 shows the MS/MS spectra of the analytes.

The capillary voltage was maintained at 0.5 kV. The cone voltage was 25V for both DXM and cyclophosphamide. The source and desolvation temperature was 130 and 400°C, respectively. The cone gas flow was kept at 55 L/h and the desolvation gas flow at 650 L/h. The collision energies were 15 and 20 V for DXM and cyclophosphamide, respectively. The dwell time was 0.2 seconds for DXM and 0.1 for cyclophosphamide. Data were processed with MassLynx 4.1 software.

### Preparation of calibration standards and quality controls

The stock solution of dexmedetomidine was prepared in 70% methanol, final concentration of 1mg/mL. From the stock DXM solution, work solutions were individually prepared (20X) in 70% MeOH, in order to obtain solutions for calibrators at 50, 250, 500, 750, 1000, 1500 and 2000 pg/mL concentrations. Solutions for low, medium and high quality control points were prepared at 150, 900 and 1600 pg/mL, respectively. Independent calibration curves were prepared with blood (haematocrit 37%) prepared stemming from the concentrated erythrocytes and plasma, both obtained from the blood reservoir of our institution. 950 μL of this blood and 50 μL of DXM solution were mixed at the different concentrations (20X).

To each circle of the Whatmann 903 cards was added 40μL of each point of the calibration curve or quality controls. Each card was allowed to dry horizontally for 6 hours at room temperature (25 ±2°C). Once dried, the cards were stored properly labelled in plastic bags with low gas permeability, accompanied by a drying material. To run the stability tests, samples were kept at room temperature, and to perform the long term stability, they were kept at -80°C, until analysed.

### Sample preparation and processing

Ten discs of 3 mm diameter filter paper were cut from two circles of filter paper (5 per cicle), using a manual punch (McGill, Inc). The disks were placed in a 1.5 mL microtube, 10 μL of cyclophosphamide were added as internal standard (100,000 pg/mL, in 70% methanol), 10 μL of ascorbic acid (10 mg/mL) and 10 μL of 10% ammonium hydroxide were added.

Subsequently, 1 mL of a mixture of ethyl acetate and dichloroethane (90:10 v/v) was added, the tube was stirred vigorously for two min in vortex and then subjected to sonication in a water bath (Ultrasons HD, JP Selecta) for 5 min. The tube was centrifuged at 12000 rpm, 5 min at 25°C.

The organic phase was transferred to a glass tube (16 x 150 mm), the sample was evaporated to dryness by means of a water bath at 40° C and a gentle flow of nitrogen (Evaporator System, Glas-Col). The dry residue was resuspended in 100 μL of acetonitrile and 0.1% formic acid in water (60:40 v/v). Subsequently, it was transferred to an autosampler vial and a 10 μL aliquot was injected into the LC-MS/MS system.

### Haematocrit effect

Blood samples were prepared with different percentages of haematocrit (25 to 45%), each sample containing 750 pg/mL of DXM. The blood samples were processed in triplicate and quantified using a calibration curve prepared with 37% haematocrit; this value was selected according to Lopez Santiago, 2016 [13]. Values varying 10% of the nominal value were accepted.

### Validation test

Validation of the method was carried out following the criteria established in the guidelines reported by Timmerman [14], which are in accordance with the international guidelines [15, 16]. The tests of matrix effect, selectivity to concomitantly administered drugs (ropivacaine, propofol, acetaminophen, fentanyl), carry-over effect, linearity, precision, accuracy, absolute recovery, limit of quantification, stability of the sample in the short and long term were performed. Additionally, stability of the samples was assayed exposed to white light or at 40°C.

### Application of the method

The Research and Ethics Committee of the National Institute of Paediatrics approved the present study, and given the registration number: 062/2014. For the applicability of this method, 6 paediatric patients with ages between 0 and 2 years, ASA I, who underwent outpatient surgical procedures were recruited. Written informed consent was obtained from the children’s parents after they were informed of the objectives and risks.

Patients who were receiving treatment with enzyme-inducing drugs, a history of arrhythmias, delayed neurological development or malnutrition were excluded from the study.

A standardised anaesthetic regimen was used with inhaled induction with a mixture of sevoflurane and oxygen. After the loss of consciousness, a venous catheter was placed for the administration of fluids and medications. Fentanyl was administered and insertion of the endotracheal or laryngeal mask with propofol was facilitated and DXM was administered as described below. Anaesthesia was maintained with sevoflurane adjusted according to the surgical procedures and patient response. At the end of the surgery, the patients were transferred to the recovery room. The vital signs were recorded during the surgery every 5 min and once the surgery was finished, every 30 min until the discharge indicated by the doctor.

### Obtaining blood samples from paediatric patients

After the placement of the intravenous catheter, the administration of dexmedetomidine was started by IV infusion diluted in physiological solution (loading dose of 1.0 μg/Kg) to 10 mL/Kg, with an infusion time of 15 min. At the conclusion of the infusion, blood samples were taken by heel puncture using an automatic and retractable lancet (contact-activated, BD Microtainer). The sampling times were: 5, 10, 15, 20, 30, 45 min and 1, 1.5, 2, 3, 5, 7, 10 and 12 h. Each patient was randomly sampled 4 times. The cards were left to dry horizontally at room temperature (25 +-2°C) for 6 h. Once dried, the cards were stored properly labelled in plastic bags with low gas permeability, accompanied by a drying material at -80°C until analysed.

### Statistics

Average (mean) data, standard deviations and coefficient of variation were processed to evaluate the validation parameters.

## Results

### Method development

During the optimization of the chromatographic conditions, different ionizing agents (0.1, 0.5 and 1% formic acid, 2 mM ammonium acetate and 5 mM ammonium format) were tested in mobile phases containing different proportions of solvents (acetonitrile or methanol). The mobile phase that provided the best ionization for the drug was 0.1% formic acid with acetonitrile (50:50 v/v).

The extraction of DXM from the samples in DBS was initially tested with acetonitrile or 100% methanol; however, these solvents rendered no extraction at all. Different solvents were tested, and the best mixture consisted in ethyl acetate and dichloroethane in a 90:10 ratio (v/v), which yielded more than 87% of DXM in DBS samples.

### Haematocrit effect

The determination of DXM, in DBS samples with different haematocrit, showed that a calibration curve with 37% haematocrit is adequate to quantify blood samples with a haematocrit of 30 to 45%; however, lower values show an underestimation in the nominal value (Table 1).

**Table 1:** Percentages of deviation obtained after quantification in triplicate of a nominal concentration (DXM 750 pg/mL), with a calibration curve prepared with a haematocrit of 37%.

Haematocrit (%)	25	30	35	40	45
Mean ± SD (pg/mL)	618.6 ± 3.4	687.5 ± 18.1	750.5 ± 7.1	753.0 ± 1.7	764.3 ± 7.5
Deviation (%)	17.5	8.3	-0.0	-0.4	-1.9

SD: Standard deviation.

### Validation

#### Linearity and sensibility

**Fig 2:**
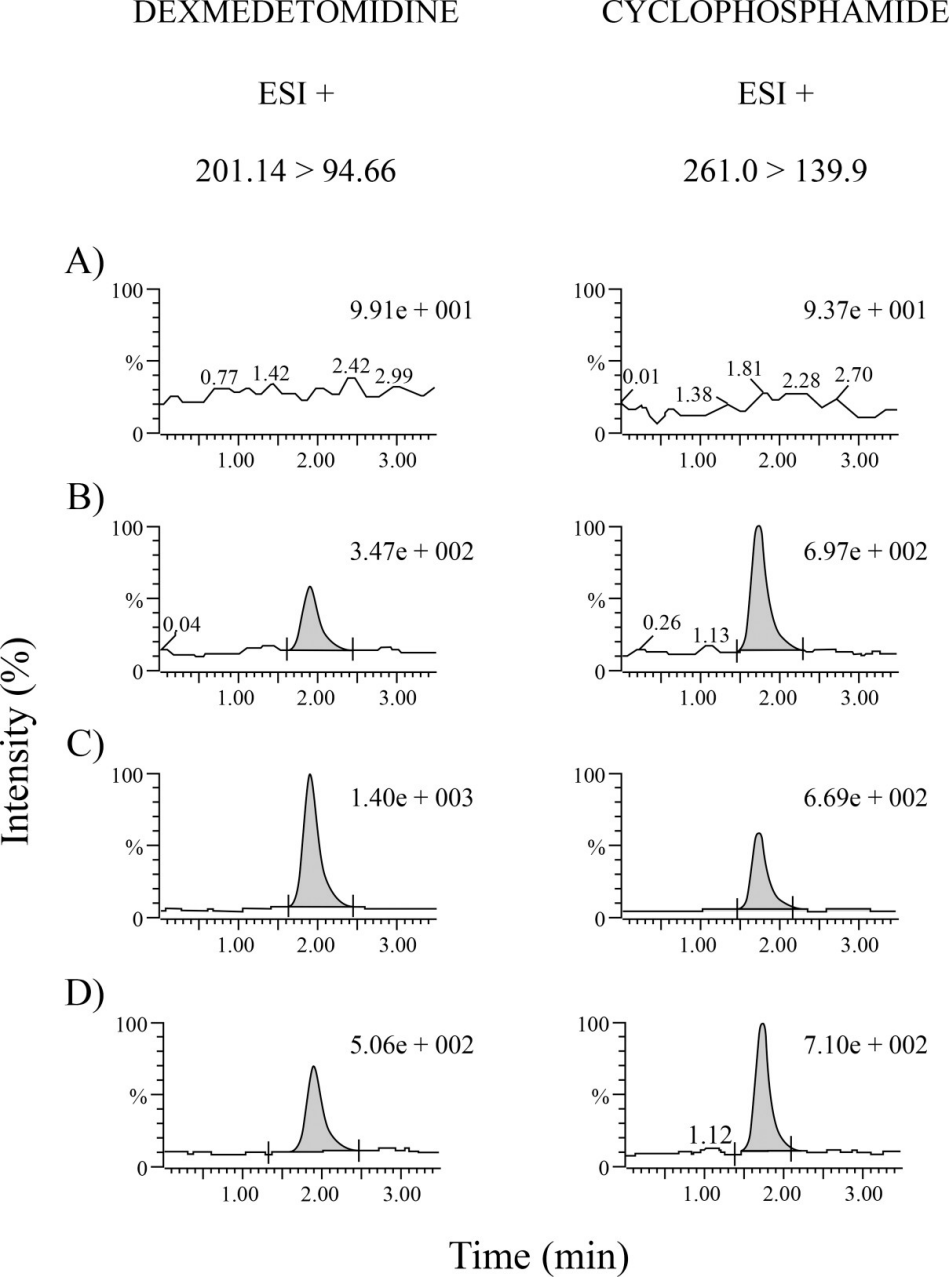
Representative chromatograms of (A) blank of dried blood spot, (B) LLOQ (50 pg/mL), (C) medium quality control (900 pg/mL) and (D) the sample from a paediatric patient.

The method is linear in the range of 50 to 2000 pg/mL determined by the analysis of 6 calibration curves, while the injection by fivefold in 3 consecutive days (n = 15) allowed to establish the lower limit of quantification in 50 pg/mL (Table 2). Fig 2 shows the typical chromatograms of the blank, low limit of quantification, a quality control and a sample from a patient.

**Table 2:** Validation tests of the analytical method by LC-MS/MS for the quantification of dexmedetomidine in dried blood spots.

	**Day 1**	**Day 2**	**Day 3**
**Limit of quantification (n = 5)**			
Mean ± SD	49.7 ± 4.7	51.0 ± 3.6	50.0 ± 3.8
CV	9.4	7.1	7.6
**Inter-day test (n = 4/day)**			
Mean ± SD	141.4 ± 6.5	159.8 ± 14.6	159.9 ± 13.7
CV	4.5	9.1	8.6
Mean ± SD	838.0 ± 38.6	1018.5 ± 20.1	905.4 ± 56.4
CV	4.6	2.0	6.2
Mean ± SD	1472.7± 78.1	1815.0 ± 2.9	1584.8 ± 107.8
CV	5.3	0.16	6.8
	**LQC (pg/mL)**	**MQC (pg/mL)**	**HQC (pg/mL)**
**Intraday test (n = 5/day)**			
Mean ± SD	149.3 ± 8.6	897.7 ± 84.9	1564.9 ± 156.9
CV	6.0	9.4	10.0
**Short-term stability (n = 3)**			
Mean ± SD	152.1 ± 10.9	1027.9 ± 18.3	1713.8 ± 51.8
CV	7.2	1.8	3.0
**Stability in autosampler (n = 3)**			
Mean ± SD	140.9 ± 11.0	948.50 ± 40.0	1714.6 ± 73.2
CV	7.8	4.2	4.3
**Light 25**°**C for 24 h (n = 4)**			
Mean ± SD	159.0 ± 5.0	957.8 ± 47.5	1645.2 ± 106.3
CV	3.1	4.9	6.4
**40**°**C for 24 h (n = 4)**			
Mean ± SD	152.3 ± 7.1	926.6 ± 30.4	1579.8 ± 13.3
CV	4.7	3.2	0.85
**Long-term stability 5 days at 25°C (n = 3)**			
Mean ± SD	148.7 ± 2.1	904.7 ±14.5	1563.2 ± 153.8
CV	1.4	1.6	9.8
**Long-term stability 187 days at -80°C (n = 4)**			
Mean ± SD	146.9 ± 7.4	883.4 ± 50.3	1514.5 ± 77.0
CV	5.0	5.6	5.0

SD: Standard deviation. CV: Coefficient of variation. Quality controls: low (LQC), medium (MQC) y high (HQC) with nominal concentrations of 150, 900 y 1600 pg/mL, respectively.

### Selectivity

By analysing samples of DBS containing drug-free blood, it was shown that there is no interference from endogenous matrix compounds. The selectivity analysis showed that the presence of drugs administered concomitantly (ropivacaine, propofol, acetaminophen, fentanyl) does not interfere with the quantification of DXM.

### Accuracy and precision

The developed method meets the criteria of accuracy and precision. The interday trial was analysed in triplicate quality controls in three consecutive days, while for the intraday test was performed by five-fold analysis on the same day of the quality controls (LQC, MQC and HQC). For all the mentioned trials the coefficient of variation was less than 10%.

### Stability

The stability of the drug was evaluated under different working conditions: in the sampler at 15°C for 8 h, room temperature for 4 h and stored in the long term (at 25°C for 5 days and at -80°C for 187 days). Additionally, was assayed stability after exposing the samples to white light at 25°C for 24 h, and at 40°C. All the determinations made reported values higher than 90% of the expected concentration, which indicates that the DXM is stable under the conditions evaluated (Table 2).

### Matrix effect

The matrix effect was determined by extracting blank samples in DBS and, at the end of the process, solutions of the analyte and the internal standard (quality controls LQC, MQC and HQC) were added, and their responses were compared with the ones of each of them separately. For each unit, a normalised matrix factor was obtained, according to the following formula.

Normalised Matrix Factor (NMF) was calculated by means of the following formula:

NMF = (Response of the analyte in the matrix/response of the internal standard in the matrix)

(Response of the analyte in solution/response of the internal standard in solution)

The acceptance criterion for the matrix effect indicates a coefficient of variation of less than 15% for the normalised matrix factor (NMF). In our trial, values of 13.5%, 13.6% and 12.8% of CV were obtained for the concentrations of the quality controls, low, medium and high, respectively. That is why we did not find matrix effect.

### Recovery

Recovery was made by comparing the response (peak area) of DBS QC (low, mid and high) sample extracts and blank DBS sample extracts post-fortified with the same concentration DBS QC. The recovery was 94.6%, 95.4% and 102.2% for quality control low, mid and high respectively, whereas for the internal standard it was 88.8%.

### Carry-over

We did not observe carry-over effect. The chromatograms of the blank samples show no interference response.

### Quantification of dexmedetomidine in paediatric patients

**Fig 3:**
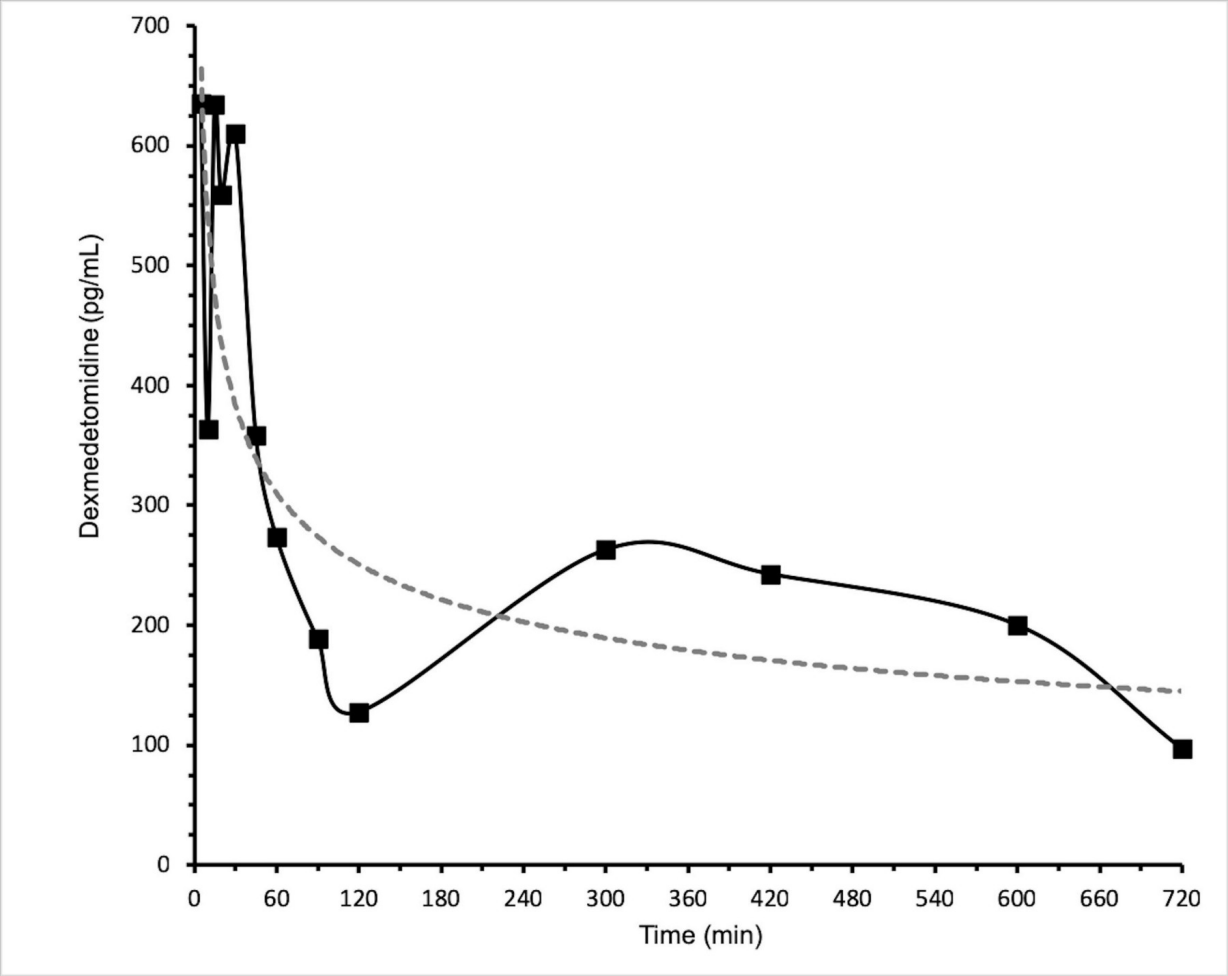
Profile of dexmedetomidine concentrations in six paediatric patients at different sampling times. Four samples were taken from each participant; the average of the determinations is shown in squares, connected with a continuous line.

Six patients (5 boys and 1 girl), less than 2 years of age were included in this study. All patients attended ambulatory surgeries and four samples were taken at different times (Table 3), in the range of the pharmacokinetic curve (5 min to 12 h). The DXM values of the 6 patients were in the range of 76.53–868.02 pg/mL (Fig 3).

**Table 3:** Demographic data and dexmedetomidine concentrations found in patients.

**Patient**	Gender	Age (months)	Weight (Kg)	Height (cm)	Surgery	DXM (pg/mL); Sampling time [min]			
1	M	1	2.8	49	Pyloric hypertrophy	355.8; [20]	356.1; [45]	244.6; [60]	170.64; [120]
2	M	9	7.4	78	Inguinal hernia	452.1; [20]	676.9; [30]	578.2; [45]	302.4; [60]
3	M	14	10.0	76	Elbow reduction	868.0; [20]	621.8; [30]	263.0; [300]	77.0; [420]
4	M	16	9.6	75	Iliac osteotomy	635.5; [5]	408.3; [420]	323.6; [600]	96.9; [720]
5	F	20	11.5	82	Rhinosepto-plasty	634.2; [15]	531.9; [30]	141.1; [45]	87.8; [90]
6	M	24	11.5	80	Hypospadias	363.7; [10]	289.9; [90]	84.6; [120]	76.5; [600]

Six children under 2 years were included in the study. All were ASA I patients, who underwent ambulatory surgeries and received the same anaesthetic regimen that included dexmedetomidine at a dose of 1 μg per Kg of body weight. Sampling times for each patient are denoted between brackets.

## Discussion

There is scarce information on important factors such as race, maturation, nutritional status and pharmacogenetics on DXM clearance in the paediatric population in general, and especially in neonates and infants. There are, also, few PK-PD studies that allow dosing recommendations for this specific age group. It is expected that with the increasingly widespread use of DXM in the paediatric population and especially neonates and infants, cases of adverse effects may increase if the clinician does not have dosage recommendations based on PK-PD studies. Thus, having a precise and minimally invasive method for quantification of DXM can be an important contribution to enhance the efficacy and safety of this drug [11].

In recent years the use of dexmedetomidine has increased in the paediatric population, even though the FDA has not approved its use in this population. One of the causes that have driven its use in infants is the ability of the drug to preserve respiratory function.

Despite some efforts to conduct pharmacokinetics and pharmacodynamics (PK-PD) clinical trials, reports of DXM in children less than two years of age are still scarce.

Thus, the use of the drug is still based on the positive response obtained in clinical practice (evidence-based medicine) and is used at the same doses recommended for adults (0.7 to 1.0 mg / Kg).

The trials of Petroz (2006), Díaz (2007), Potts (2008), Su (2010) and Tobias (2011) [17–21] include paediatric patients with heart diseases and/or in intensive care units, to which doses were administered DXM at the doses used in adults. These studies estimated some pharmacokinetic parameters (area under the curve, clearance, volume of distribution), however, an adequate dosage regimen was not determined.

Vilo et al [22] performed the pharmacokinetics of dexmedetomidine in patients ASA I and II submitted to imaging studies; In this trial, each participant was taken from 4 to 11 blood samples by venepuncture (volume of 1 mL per sample).

In this study, we developed and validated a method that allows obtaining samples for the quantification of dexmedetomidine concentrations with a drop of blood and minimal discomfort for the patient, especially in neonates and infants. The application of this method can overcome one of the limitations to accomplish DXM PK-PD studies in this population of patients. The use of DBS is a great advantage, the sampling is less invasive and painful than venepuncture, the volume collected is one drop of blood (approximately 40 μL), which facilitates multiple taking for pharmacokinetic studies and decreases the possibility of complications such as phlebotomy-induced anaemia [23, 24]. In neonates, it has been reported that taking multiple blood samples increases the risk of complications (10 mL of blood may contain up to 4 mg of iron [25, 26], with a weight of 2.7–3.6 Kg, a maximum of 2.5 mL of blood per dose has been established [27], higher volumes could cause complications in patients given the circulating blood volume (80 mL/Kg) [28].

Previous reports to quantify DXM have used methods of drug extraction such as direct protein precipitation [29], solid phase extraction [30–34] or liquid-liquid [35–37]. These reports require volumes of 100 to 1000 μL of plasma. The work of Moosavi and colleagues (2018) uses 50 μL of plasma with a limit of quantification of 500 pg/mL, in the present study, only 40 μL of blood (equivalent to one drop) is used and it has a limit of quantification of 50 pg/mL.

In addition, our analysis showed that DXM was stable in the biological matrix (dried blood) embedded in the Whatmann 903 filter paper for at least 187 days at -80°C and for 5 days at room temperature. This feature facilitates the transport and storage of the samples and avoids the need to maintain a cold chain for the correct conservation of the sample.

The method developed was adequate to quantify DXM in patients younger than two years, the concentrations were in the range of 76.53 to 868.00 pg/mL. To date only Vilo (2008) has conducted a study with patients less than 2 years (ASA I or ASA II); this trial included 8 patients, aged 28 days to 23 months, who were administered a dose of 1 μg/Kg. The reported concentrations (adjusted to the Akaike model) were in the range of 40 to 500 pg/mL, which are similar to those reported in the present study (76 to 858 pg/mL).

Likewise, the profile of the average values of our patients showed the characteristic redistribution of dexmedetomidine (between 5 and 10 min), this redistribution is not observed in the profile shown by Vilo.

The concentrations shown in other studies, which include patients with heart disease or intensive therapy, are different from those of the present study; this may be due to factors such as the administration of concomitant drugs, the degree of disease and its underlying pathology. However, it is noteworthy that in all previous studies and in the present a high variability among individuals is observed, which prompts to perform a complete pharmacokinetic study that includes patients from 0 to 2 years to define the optimal dose of the drug.

## Conclusion

The method developed in this work is linear, reproducible, exact and selective; it also offers the advantage of being simple and very non-invasive, since it allows quantifying the drug in blood samples as small as one drop. Therefore, it will be a useful tool to carry out PK-PD studies, in such a way that the therapeutic interval can be defined, dosage regimens can be designed and the possible toxicity of the drug determined, which has not been defined in paediatric patients. This method is recommended for paediatric clinical practice and in general for any patient receiving DMX.

The use of this method can promote an important advance in the knowledge of the pharmacokinetics and pharmacodynamics of dexmedetomidine, especially in neonates and infants, contributing to greater efficacy and safety in its application in these patients.

## Supporting information

S1 File Data set of all determinations. Document contains the data and the statistics shown in the present manuscript.
(XLSX)
